# Flow diversion for compressive unruptured internal carotid artery aneurysms with neuro-ophthalmological symptoms: a systematic review and meta-analysis

**DOI:** 10.1136/jnis-2022-019249

**Published:** 2022-08-02

**Authors:** Daniel P O Kaiser, Ani Cuberi, Jennifer Linn, Matthias Gawlitza

**Affiliations:** 1 Department of Neuroradiology, University Hospital Carl Gustav Carus, Dresden, Germany; 2 EKFZ for Digital Health, Dresden University of Technology, Dresden, Germany; 3 Department of Radiology, University Hospital Carl Gustav Carus, Dresden, Germany

**Keywords:** Complication, Aneurysm, Flow Diverter, Stent

## Abstract

**Background:**

Data on the safety and efficacy of flow diverters (FD) for the treatment of unruptured internal carotid artery (ICA) aneurysms with compressive neuro-ophthalmological symptoms (NOS) are scarce and comprise mainly small case series.

**Methods:**

We performed a search of three databases and included series with ≥10 patients, with unruptured aneurysms of the ICA and NOS, treated with FD. Random-effects analysis of treatment results and safety was performed.

**Results:**

A total of 22 studies reporting on 594 patients were included. Pooled proportions of NOS recovery, improvement, transient and permanent worsening were: 47.4% (95% CI 35.0% to 60.1%); 74.5% (95% CI 67.9% to 80.2%); 7.1% (95% CI 3.3% to 14.7%); and 4.9% (95% CI 3.2% to 7.4%), respectively. Rates of complete recovery and improvement in patients with isolated visual symptoms were 30.6% (95% CI 12.5% to 57.7%) and 56.6% (95% CI 42.3% to 69.9%). Isolated oculomotor symptoms recovered completely in 47.8% (95% CI 29.9% to 66.3%) and improved in 78% (95% CI 69.2% to 84.9%). Morbidity occurred in 5% (95% CI 2.8% to 9%) and mortality in 3.9% (95% CI 2% to 7.5%) of patients. An increased likelihood of symptom improvement was observed when treatment was performed early (<1 month) after symptom onset (OR=11.22, 95% CI 3.9% to 32.5%).

**Conclusion:**

Flow diversion promotes recovery or improvement of compressive symptoms in a large proportion of patients but is associated with significant rates of morbidity and mortality. Transient and permanent NOS worsening is not uncommon. Early treatment is of utmost importance, as it increases the likelihood of symptom improvement more than 10-fold.

**WHAT IS ALREADY KNOWN ON THIS TOPIC**

- There are limited data in the literature on flow diversion for unruptured internal carotid artery (ICA) aneurysms with compressive neuro-ophthalmological symptoms.

**WHAT THIS STUDY ADDS**

- This meta-analysis provides a comprehensive overview of the efficacy and safety of flow diversion in this specific patient population.

**HOW THIS STUDY MIGHT AFFECT RESEARCH, PRACTICE OR POLICY**

- Flow diversion is an effective and valuable treatment strategy for patients with compressive ICA aneurysms and neuro-ophthalmological symptoms. However, it is important to treat patients early after symptom onset and to be aware of the non-negligible morbidity and mortality rate.

## Introduction

Aneurysms of the internal carotid artery causing mass effect and neuro-ophthalmological symptoms (NOS) by compression of the cranial nerves (CN) are a rare pathology. Visual impairment or diplopia induced by CN palsy are disabling symptoms and of high relevance for the patient’s quality of life. Aneurysms inducing compression-related symptoms are often large and/or rapidly growing lesions.^1^ Intrasaccular coil embolization, parent artery occlusion (PAO)—either with or without extracranial–intracranial bypass surgery—or aneurysm clipping have been studied for the management of these lesions.^2–5^ Flow diverters (FD) promote aneurysm collapse and healing, thus reducing the mass effect, while preserving the vessel patency.^6^ To date, the literature on the use of FD in internal carotid artery (ICA) aneurysms causing compressive NOS is still scarce. The present study aims to provide a systematic review of the literature and meta-analysis of this treatment method, aiming to provide physicians involved in aneurysm treatment with a realistic pooled estimate of treatment efficacy and safety. Moreover, we sought to investigate the relevance of time lapse from symptom onset to treatment on the rates of symptom improvement.

## Methods

### Ethics statement

Approval of the ethics committee was not required for this study as only published primary studies were analyzed. This study was not registered.

### Search strategy

The senior author independently reviewed the literature on PubMed, Scopus, and Web of Science, using a predefined search algorithm (detailed in the online supplemental material). We searched titles, abstracts, and keywords. Duplicates were removed, titles were screened and abstracts were reviewed. Second, if potentially eligible for this analysis, the full text paper was retrieved and reviewed thoroughly. The first and the senior author extracted the data and entered them into a predefined data sheet; discrepancies were solved by consensus.

10.1136/jnis-2022-019249.supp1 Supplementary data

### Inclusion criteria

We included series reporting on ≥10 patients with (1) an unruptured intracranial aneurysm of the ICA, with (2) a compressive effect on the oculomotor nerves and/or the optic pathway, considered responsible for ocular symptoms—that is, (3) cranial neuropathy affecting the CN III, IV, and VI (alone or in combination) and/or (4) visual impairment due to compressive optic neuropathy. Treatment was (5) with flow diversion alone or in conjunction with coil embolization.

### Data extraction and outcome measures

The objectives of this meta-analysis were to summarize the clinical and anatomical efficacy (compressive symptom improvement or complete recovery and aneurysm occlusion) and the safety (treatment-related thromboembolic and hemorrhagic complications with permanent deficit or death) of flow diversion for treatment of compressive ICA aneurysms with neuro-ophthalmological symptoms. Secondary endpoints were the rates of transient and permanent symptom worsening and the impact of time from symptom onset to treatment on the symptom improvement rate.

We extracted, with as much detail as possible, patient-, aneurysm-, and treatment-specific data from the original articles. If necessary and possible, values were recalculated from individual patient data provided in the publications—for example, in tables or the appendix. Data from the series of Boulouis *et al* 7 were calculated from the original raw dataset.

We extracted data on isolated visual or oculomotor symptoms, or a combination of both. CN deficits at follow-up were graded as ‘complete recovery’, ‘partial recovery’, and ‘permanent worsening’. The sum of patients with ‘complete recovery’ and ‘partial recovery’ was defined as ‘improvement’. Articles were furthermore screened for signs of ‘transient worsening’ of CN deficits after flow diversion.

Morbidity was defined as any neurological deterioration of the patient’s status (except worsening of NOS), related to presumed hemorrhagic or ischemic complications.

Aneurysm occlusion grades were extracted at last follow-up using the widely accepted classification: ‘aneurysm remnant’, ‘neck remnant’, and ‘complete occlusion’.^8^ ‘Neck remnant’ and ‘aneurysm remnant’ were grouped as ‘incomplete occlusion’. When an alternative grading scale was used,^9^ only grade D was considered ‘complete occlusion’.

### Statistical analysis

The analysis was performed using primarily R Studio (R Studio, Boston, USA, version 2022.02.2) with the metafor^10^ and meta^11^ packages. Random-effect analyses were performed after logit transformation. Results are presented as percentage and 95% CI. I^2^ statistic and Q-test were used to assess study heterogeneity. Publication bias was assessed by visual inspection of funnel plots and with Egger’s unweighted regression test. Pooled effects of early versus late treatment (ie, within 1 month vs beyond 1 month after symptom onset) were calculated using the RevMan 5 software package,^12^ applying random-effects analysis. We performed an additional random-effect meta-regression, studying the effect of mean/median patient age, length of follow-up, and study size as moderators on the effect size of complete NOS recovery and improvement using SPSS Statistics 28 (IBM, Armonk, USA).

## Results

### Study inclusion

Literature search was performed on March 21, 2022. After removal of duplicates and screening of titles and abstracts, we sought for the original articles of 82 publications.^7 13–93^ After completion of literature review and data extraction and before closing the database, the literature search was repeated on PubMed only on May 22, 2022, using the above-mentioned search string to identify additional potentially eligible articles. Two papers published in the meantime were identified.^94 95^ Four papers published in Chinese in Chinese journals could not be retrieved.^61 66 69 70^ Thus, 80 papers were screened for eligibility. Detailed information on publication inclusion and exclusion are depicted in online supplemental figure 1 and online supplemental table 1.

### Descriptive results

Altogether, 22 studies were included, encompassing 594 patients treated with flow diversion for an unruptured intracranial aneurysm of the ICA and compression-related neuro-ophthalmological symptoms. An overview of the included studies is shown in table 1. Online supplemental table 2 depicts patients demographics and aneurysm characteristics. Data on isolated visual or oculomotor symptoms were extracted for 149 and 293 patients, respectively. All relevant data are shown in the online supplemental material. Dedicated neuro-ophthalmological follow-up protocols were mentioned in three publications only.^37 46 95^ Neuro-ophthalmological outcomes are depicted in table 2 and in online supplemental tables 3 and 4. Online supplemental table 5 summarizes the neurological complications and anatomical results.

**Table 1:** Study overview

Study	Year	Design	Pts/pts with NOS* (%)	Flow diversion devices used	Dedicated NOS F/U	Comments
Yu *et al* 22	2012	PM	143/14 (10.2%)	PED	NR	
Szikora *et al* 28	2013	PS	29/16 (55.2%)	PED, Silk	NR	Overlap with PUFS/Sahlein *et al* 46
O’Kelly *et al* 26	2013	PM	97/36 (37.1%)	PED	No	Data discrepancy in the manuscript, data from text were used
Moon *et al* 35	2014	RS	20/19 (95%)	PED	NR	Data recalculated
Tanweer *et al* 37	2014	RS	41/19 (46.3%)	PED	Yes	Data discrepancy in the manuscript, data from text were used
Zanaty *et al* 34	2014	RS	157/51 (33.8%)	PED	NR	
Zhou *et al* 38	2014	PS	28/11 (39.3%)	Tubridge	NR	
Puffer *et al* 31	2014	PM	44/24 (54.5%)	PED, Silk, Surpass	NR	Overlap with PUFS/Sahlein *et al.* 46. Data recalculated
Sahlein *et al* 46	2015	PM	108/39 (36.1%)	PED	Yes	Only patients with initial aneurysm-induced NOS included
Zanaty *et al* 45	2015	RS	44/12 (27.3%)	PED	NR	
Breu *et al* 49	2016	RS	28/10 (53.7%)	Silk, PED	NR	Data recalculated
Kim *et al* 53	2016	RM	45/18 (40%)	PED	NR	
Brown *et al* 54	2016	RM	45/45 (100%)	PED	NR	Overlap with PUFS/Sahlein *et al* 46
Miyachi *et al* 64	2017	RM	24/18 (75%)	PED	No	Data recalculated
Silva *et al* 71	2018	RS	115/21 (18.3%)	PED	NR	
Oishi *et al* 73	2018	RS	100/38 (38%)	PED	NR	
Yan *et al* 79	2019	RS	126/50 (39.7%)	PED	NR	Data recalculated
Wang *et al* 76	2019	RS	22/22 (100%)	PED	No	Data recalculated
Boulouis *et al* 7	2021	RM	55/54† (98.2%)	PED, Silk, p64, Derivo, Surpass	No	Raw data access
Fujii *et al* 93	2022	RS	112/29 (25.9%)	PED	NR	Potential overlap with Oishi *et al* 73
Xu *et al* 95	2022	RS	189/29 (15.3%)	PED	Yes	
Lee *et al* 94	2022	RS	49/28 (57.1%)	NR	NR	

*NOS=neuro-ophthalmological symptoms induced by internal carotid artery aneurysm, treated with flow diversion.

†One patient treated with parent vessel occlusion excluded from the original publication.^107^

F/U, follow-up; NR, not reported; PED, Pipeline embolization device; PM, prospective multicenter; PS, prospective single-center; RM, retrospective multi-center; RS, retrospective single-center.

**Table 2:** Overall neuro-ophthalmological outcomes

Study	Symptom onset to treatment (mean±SD)	Patients with NOS* and F/U	NOS F/U (mean±SD)	Complete recovery	Partial recovery	Improvement (complete and partial recovery)	No change	Transient worsening	Permanent worsening
Yu *et al* 22	NR	13	3.5 months (median)	10/13 (76.9%)	0 (0%)	10/13 (76.9%)	3/13 (23.1%)	0/13 (0%)	0 (0%)
Szikora *et al* 28	NR	16	NR	10/16 (62.5%)	5/16 (31.3%)	15/16 (93.8%)	0/16 (0%)	3/16 (18.8%)	1/16 (6.3%)
O’Kelly *et al* 26	NR	27	NR	12/27 (44.4%)	6/27 (22.2%)	18/27 (66.7%)	9/27 (33.3)	2/27 (7.4%)	0/27 (0%)
Moon *et al* 35	50.4 weeks (mean)	19	9.7±6.3 months	3/19 (15.8%)	11/19 (57.9%)	14/19 (73.7%)	5/19 (26.3%)	2/19/10.5%)	0/19 (0%)
Tanweer *et al* 37	NR	19	NR	NR	NR	16/19 (84.2%)	3/19 (15.8%)	0/19 (0%)	0/19/0%)
Zanaty *et al* 34	NR	51	NR	36/51 (70.6%)	11/51 (21.6%)	47/51 (92.2%)	4/51 (7.8%)	0/51 (0%)	0/51 (0%)
Zhou *et al* 38	NR	11	NR	4/11 (36.4%)	4/11 (36.4%)	8/11 (72.7%)	3/11 (27.3%)	2/11 (18.2%)	0/11 (0%)
Puffer *et al* 31	NR	20	10.3±7.6 months	18/20 (90%)	0/20 (0%)	18/20 (90%)	2/20 (10%)	2/20 (10%)	0/20 (0%)
Sahlein *et al* 46	NR	39	6 months	2/39 (5.1%)	22/39 (56.4%)	24/39 (61.5%)	13/39 (33.3%)	0/39 (0%)	2/39 (5.1%)
Zanaty *et al* 45	NR	12	NR	9/12 (75%)	3/12 (25%)	12/12 (100%)	0/12 (0%)	0/12 (0%)	0/12 (0%)
Breu *et al* 49	NR	9	NR	NR	NR	6/9 (66.7%)	3/9 (33.3%)	0/9 (0%)	0/9 (0%)
Kim *et al* 53	NR	18	NR	NR	NR	15/18 (83.3%)	3/18 (16.7%)	15/18 (83.3%)	0/18 (0%)
Brown *et al* 54	11 within 4 weeks, 27 beyond	45	8.4 months	19/45 (42.2%)	11/45 (24.4%)	30/45 (66.6%)	14/45 (31.1%)	0/45 (0%)	1/45 (2.2%)
Miyachi *et al* 64	NR	18	3–6 months	6/18 (33.3%)	10/18 (55.6%)	16/18 (88.9%)	2/18 (11.1%)	8/18 (44.4%)	0/18 (0%)
Silva *et al* 71	NR	15	NR	NR	NR	14/15 (93.3%)	1/15 (6.7%)	0/15 (0%)	0/15 (0%)
Oishi *et al* 73	NR	38	13.5 months	NR	NR	18/38 (47.4%)	17/38 (44.7%)	3/38 (7.9%)	0/38 (0%)
Yan *et al* 79	NR	50	6–33 months	31/50 (62%)	12/50 (24%)	43/50 (86%)	NR	NR	NR
Wang *et al* 76	11 within 4 weeks, 11 beyond	21	25.5±1.7 months	6/21 (28.6%)	6/21 (28.6%)	12/21 (57.1%)	5/21 (23.8%)	0/21 (0%)	4/21 (19%)
Boulouis *et al* 7	16.2±7.6 weeks	54	13.2±10.4 months	19/54 (35.2%)	18/54 (33.3%)	37/54 (68.5%)	10/54 (18.5%)	0/54 (0%)	3/54 (5.6%)
Fujii *et al* 93	NR	29	36 months	NR	NR	20/29 (69%)	7/29 (24.1%)	0/29 (0%)	2/29 (6.9%)
Xu *et al* 95	8 weeks (median)	26	NR	NR	NR	20/26 (76.9%)	6/26 (23.1%)	0/26 (0%)	0/26 (0%)
Lee *et al* 94	NR	28	NR	NR	NR	15/28 (53.6%)	NR	NR	NR

*NOS=neuro-ophthalmological symptoms induced by internal carotid artery aneurysm, treated with flow diversion.

F/U, follow-up; NR, not reported.

### Pooled proportions

**Figure 1:**
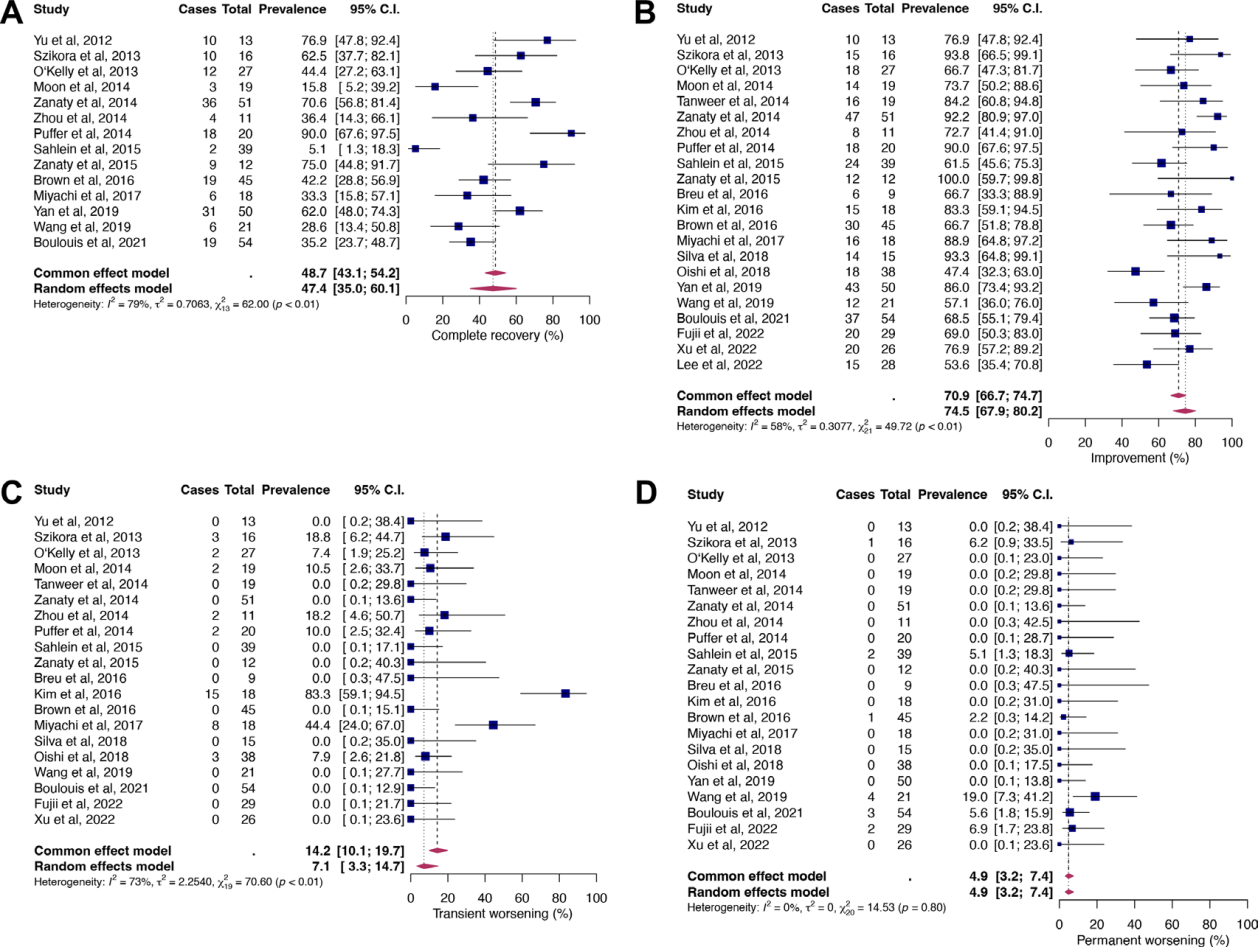
Forest plots for the proportions of complete recovery (A), improvement (B), transient (C), and permanent worsening (D).

Random-effect modeling analysis of NOS (figure 1) showed pooled rates of 47.4% (95% CI 35.0% to 60.1%) for complete recovery, 74.5% (95% CI 67.9% to 80.2%) for improvement, 7.1% (95% CI 3.3% to 14.7%) for transient, and 4.9% (95% CI 3.2% to 7.4%) for permanent symptom worsening. For all parameters except permanent worsening (I^2^=0%, p=0.8), significant moderate to substantial study heterogeneity (I^2^ between 58% and 79%) was detected (see figure 1). Visual inspection of funnel plots (online supplemental figure 2) and results of Egger’s test revealed significant asymmetry for the parameters improvement (p=0.03, online supplemental figure 2B), transient (p<0.0001, online supplemental figure 2C) and permanent worsening (p<0.0001, online supplemental figure 2D). No significant asymmetry was observed for complete recovery (online supplemental figure 2A; p=0.91).

**Figure 2:**
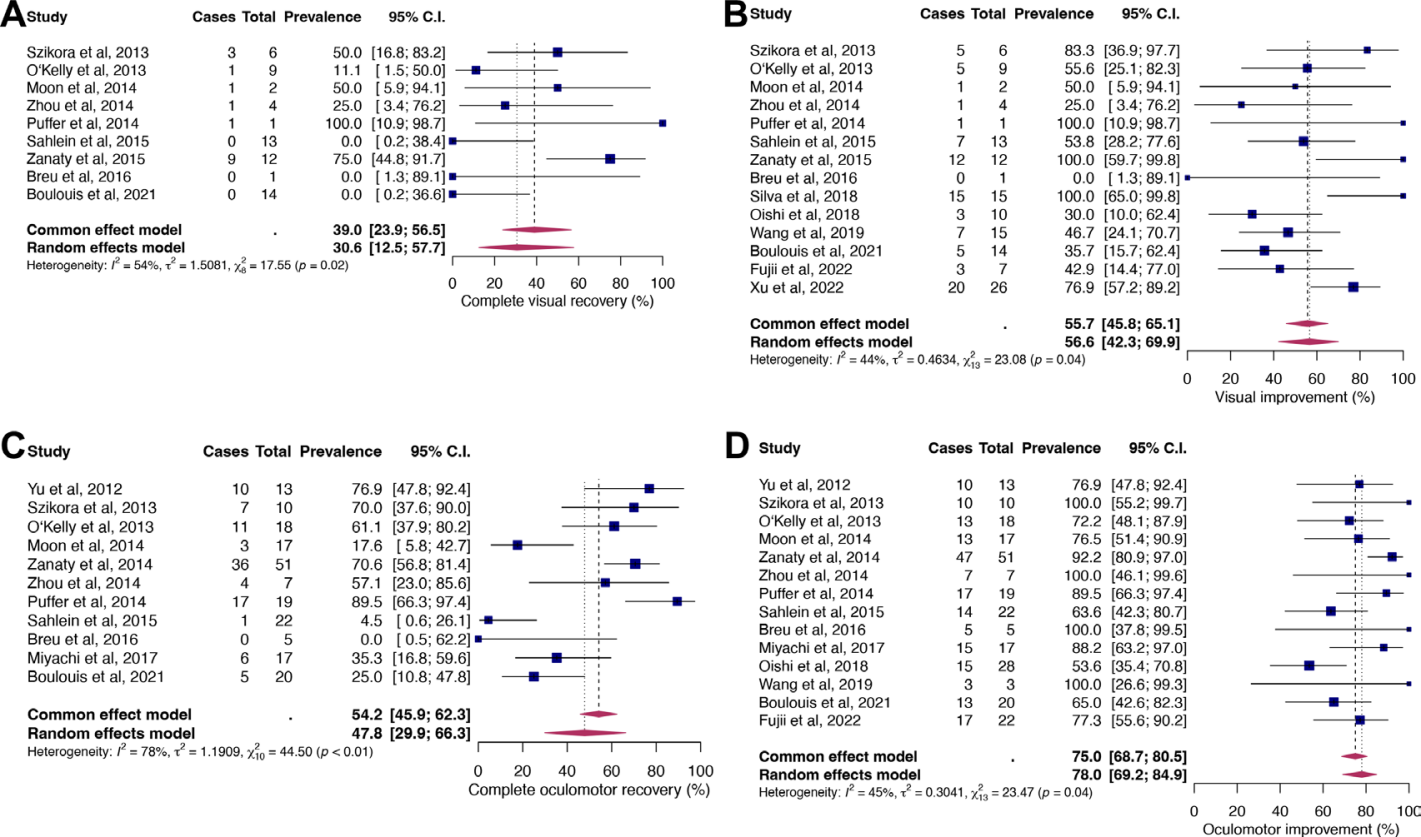
Forest plots for the proportions of complete visual recovery (A) and improvement (B), and complete oculomotor recovery (C) and improvement (D).

Pooled rates of complete recovery and improvement in patients with isolated visual symptoms (figure 2A,B) were 30.6% (95% CI 12.5% to 57.7%) and 56.6% (95% CI 42.3% to 69.9%), respectively. Isolated oculomotor symptoms (figure 2C,D) recovered completely in 47.8% (95% CI 29.9 to 66.3) and improved in 78% (95% CI 69.2 to 84.9). All parameters demonstrated significant moderate to substantial heterogeneity (I^2^ between 44% and 78%, p<0.05). Funnel plots (online supplemental figure 3) and Egger’s test revealed publication bias only for the parameter oculomotor improvement (p=0.006; other p values >0.05).

The pooled estimate of complete aneurysm occlusion at last follow-up was 68.6% (95% CI 58.8% to 77%). No significant heterogeneity or publication asymmetry was observed (Egger’s test p=0.12; online supplemental figure 4).

**Figure 3:**
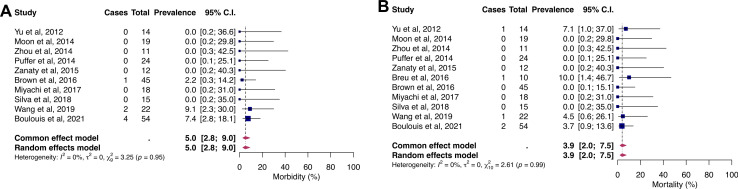
Forest plots for the proportions of morbidity (A) and mortality (B).

The pooled proportions of morbidity and mortality were 5% (95% CI 2.8% to 9%) and 3.9% (95% CI 2% to 7.5%), as shown in figure 3. Neither significant heterogeneity nor asymmetry (online supplemental figure 5; Egger’s test p>0.05) were detected.

### Early versus late treatment

**Figure 4:**
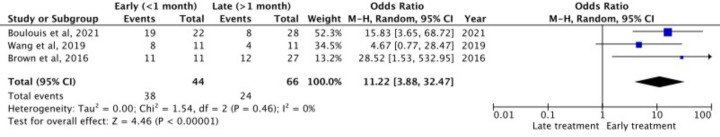
Forest plots for the effect of early (within 1 month) and delayed (>1 month) treatment on symptom improvement.

For a subset of 110 patients, information on time lapse from symptom onset to treatment were available. Random-effects analysis showed an increased likelihood of symptom improvement when treatment was performed early (ie, within 1 month) after symptom onset (OR=11.22, 95% CI 3.9% to 32.5%). The respective Forest plot is shown in figure 4, no relevant heterogeneity was detected.

### Influence of patient age, length of follow-up, and study size on neuro-ophthalmological outcome

Meta-regression revealed a significant effect of patient age on improvement of NOS (p=0.006; R^2^=100%) and a non-significant association with complete NOS recovery (p=0.126; R^2^=61.6%), as is shown in online supplemental figure 6A,B. No relevant effect on NOS complete recovery and improvement was detected when using the length of follow-up (in months) and the study size as moderators (online supplemental figure 6C–F).

## Discussion

Our meta-analysis of 594 patients treated with FD for ICA aneurysm with compressive NOS is the first to give a global overview on the literature for this specific patient population and treatment technique. Forty-eight percent of the patients treated with flow diversion recovered completely from their initial deficit and almost 75% showed improvement of compressive symptoms. Transient and permanent worsening occurred in 7.1% and 4.9% of patients, respectively. Complications were not uncommon, however, with morbidity occurring in 5% and mortality in 3.9% of patients. Complete recovery and improvement were less common in patients with isolated visual symptoms (30.6% and 56.6%), than in those with with isolated oculomotor symptoms (47.8% and 78%). Early treatment of symptomatic aneurysms with compressive symptoms seems to be essential: our analysis suggests that the likelihood of symptom improvement increases more than 10-fold if treatment is performed within the first month.

Alternative treatment methods differ, depending on the location of the aneurysm. Extradural aneurysms have historically been treated mostly with PAO only or in conjunction with an extracranial–intracranial bypass surgery in cases of a negative test occlusion. A meta-analysis from 2015 found an improvement in mass effect in 83% of patients treated with PAO only, which is comparable to the present data.^96^ Also, the rates of morbidity and mortality of PAO only (7% and 4%) were comparable with the current data for flow diversion but they increased to 11% and 7% when an additional bypass was needed for PAO.^96^ Interestingly, the authors found also that selective coil embolization of the culprit aneurysm leads to symptom improvement in 72% but is associated with a high re-treatment rate for 18%, given that large and giant aneurysms often recur after coil embolization.^97^ In our interpretation of the data, selective coiling of compressive extradural aneurysms is not an expedient treatment, as it is most probably not durable and aneurysm recurrence remains in many instances only a question of time. But also, in modern times PAO remains a valuable option, particularly if the vessel can be sacrificed without prior bypass surgery. The increased odds of complications with this surgical procedure may, however, favor flow diversion for patients for whom an occlusion test has failed.

Compressive intradural aneurysms, arising on the distal intracranial ICA were in the past mainly treated with microsurgical clipping or selective coil embolization. A meta-analysis of the treatment of paraclinoid aneurysms^98^ found that vision improved in 58% of patients after clipping and 49% after coiling. Vision worsened in 11% of patients after clipping and 9% after coiling. Interestingly, 71% vision improvement and 5% worsening were described in that analysis for FD. For compressive aneurysms of the posterior communicating artery segment, microsurgical clipping is an even more well-studied and valid option. Meta-analyses conducted for ruptured and unruptured aneurysms found higher rates of symptom recovery/improvement in patients treated surgically compared with intrasaccular coiling.^3 99^ Additionally, a large proportion of posterior communicating artery aneurysms develop NOS in the setting of rupture and are thus not eligible for flow diversion.^100^ The observation that the odds of NOS improvement and possibly also of complete recovery tend to increase with patient age is surprising, as nerve regeneration is known to be delayed and less effective in the aging individual.^101^ Accordingly, in a recent study increasing age was associated with incomplete recovery, and patients recovering completely were significantly younger than those who showed incomplete recovery only.^7^

The present meta-analysis underpins the importance of timely treatment, as the likelihood of symptom improvement increases more than 10-fold if treatment is performed within the first month. Prompt diagnosis and treatment of these patients is thus paramount and delays should be avoided, also when the aneurysm is unruptured.

The pooled rates of morbidity and mortality were 5% and 3.9%, respectively, which is higher than the findings of PUFS (morbidity/mortality rate of 5.6%),^102^ but comparable to the International Retrospective Study of the Pipeline Embolization Device (IntrePED). In that registry, neurologic morbidity/mortality was observed in 9.2% of patients with unruptured aneurysms of the ICA measuring more than 10 mm.^103^ As recent studies have shown that the risk of morbidity/mortality increases more than threefold per decade of age,^7 104^ we conclude that treatment with FD for compressive ICA aneurysms in elderly patients should be considered only after careful consideration of the risk–-benefit ratio. The fact that chances of complete symptom recovery may decrease with increasing age, fusiform aneurysm morphology, and a longer delay between the onset of ocular symptoms and endovascular treatment should be taken into account. This is important in particular for extradural aneurysms, which pose a negligible statistical risk of hemorrhage in the elderly patient.^105^

The pooled rate of complete occlusion (68.6%) is comparable to published data in the literature. While complete occlusion was observed in 86.8% in PUFS after 12 months,^102^ which should be seen as highly selected patient sample, complete occlusion at 12 months was described in 75.8% of aneurysms in a single-centre series of 1000 aneurysms treated with the PED.^106^

Our meta-analysis has some limitations. It is inherently flawed by the fact that many included publications are retrospective, often single-center case series. Moreover, earlier series on FD (for example^34 35 37 45^) bear the risk of overlap with the subset analysis of patients with NOS in the PUFS study by Sahlein *et al* 46; some studies explicitly stated that patients had been at least partly included in PUFS.^28 31 54^ A small number of double inclusions in this meta-analysis must thus be assumed. Another limitation is that in many studies, no specific demographic and procedural details were given for the subset of patients with NOS, as they were described as a fraction of a larger study on FD use for ICA aneurysms. Overall, the extracted data are characterized by substantial study heterogeneity and signs of publication bias and only in a minority of publications was specialized neuro-ophthalmological follow-up carried out.

## Conclusion

Flow diversion for compressive ICA aneurysms with NOS leads to recovery or improvement of compressive symptoms in a large proportion of patients and is a valuable treatment strategy—in particular, if sacrifice of the parent vessel is not possible. However, it is associated with significant rates of morbidity and mortality, and transient or permanent NOS worsening is not uncommon. Early detection and treatment of compressive aneurysms is paramount, as treatment within the first month from symptom onset increases the likelihood of symptom improvement more than 10-fold. The present literature is characterized by significant heterogeneity and publication bias and only a minority of publications specified dedicated neuro-ophthalmological follow-up investigations. Controlled data should thus be obtained in the future, potentially also providing solid evidence on which treatment should be chosen for which patient.

10.1136/jnis-2022-019249.supp2 Supplementary data

## Data Availability

All data relevant to the study are included in the article or uploaded as supplementary information.
