# Supplementary material for: Flow diversion for compressive unruptured internal carotid artery aneurysms with neuro-ophthalmological symptoms: a systematic review and meta-analysis
Source: J Neurointerv Surg. 2022 Aug 2;15(9):892–7. doi: 10.1136/jnis-2022-019249 (PMC10447391; doi:10.1136/jnis-2022-019249)
Supplement: Supplementary data [file jnis-2022-019249supp001.pdf]

SUPPLEMENTAL MATERIAL

Search algorithm

(“Aneurysm”[MeSH] OR “aneurysm”[tiab]) AND (“Carotid Artery, Internal”[MeSH] OR “Ophthalmic Artery”[MeSH] OR “ophthalmic”[tiab] OR “paraclinoid”[tiab] OR “clinoidal”[tiab] OR “superior hypophyseal”[tiab] OR “cavernous”[tiab] OR “petrous”[tiab] OR “visual”[tiab] OR “compressive”[tiab] OR “compression”[tiab] OR “cranial nerve”[tiab]) AND (“flow diversion”[tiab] OR “flow diverter”[tiab] OR “flow diverter”[tiab] OR “flow diverting” OR “Pipeline embolization device” OR Silk OR Fred OR P64 OR Surpass OR “Pipeline flex” OR Tubridge). [tiab] and [MeSH] arguments were removed for literature search on Scopus and Web of Science.

Supplemental Table 1: Study selection

No.	Year	First Author	Journal	DOI	Inclusion	Reason for exclusion
1	2010	Lubicz	Stroke	10.1161/STROKEAHA.110.589911	No	Less than 10 patients with cranial nerve palsies treated by flow diversion
2	2010	Szikora	AJNR. American journal of neuroradiology	10.3174/ajnr.A2023	No	Less than 10 patients with cranial nerve palsies treated by flow diversion
3	2011	Leonardi	Interventional neuroradiology	10.1177/159101991101700305	No	At least 10 eligible patients, but insufficient information on clinical symptoms and follow-up
4	2011	Nelson	AJNR. American journal of neuroradiology	10.3174/ajnr.A2421	No	No detailed information on cranial nerve palsies
5	2012	Lanzino	AJNR. American journal of neuroradiology	10.3174/ajnr.A3207	No	No detailed information on cranial nerve palsies
6	2012	Puffer	Journal of neurosurgery	10.3171/2011.11.JNS111612	No	No detailed information on cranial nerve palsies
7	2012	Briganti	Neuroradiology	10.1007/s00234-012-1047-3	No	No detailed information on cranial nerve palsies
8	2012	Berge	American Journal of Neuroradiology	10.3174/ajnr.A2907	No	At least 10 eligible patients, but insufficient information on clinical symptoms and follow-up
9	2012	Kan	Neurosurgery	10.1227/NEU.0b013e31827060d9	No	At least 10 eligible patients, but insufficient information on clinical symptoms and follow-up
10	2012	Yu	Radiology	10.1148/radiol.12120422	Yes	
11	2013	Malatesta	La Radiologia medica	10.1007/s11547-013-0944-9	No	Less than 10 patients with cranial nerve palsies treated by flow diversion
12	2013	Szikora	AJNR. American journal of neuroradiology	10.3174/ajnr.A3547	Yes	
13	2013	O'Kelly	AJNR. American journal of neuroradiology	10.3174/ajnr.A3224	Yes	
14	2013	Colby	Journal of neurointerventional surgery	10.1136/neurintsurg-2012-010299	No	No detailed information on cranial nerve palsies
15	2013	Toma	British Journal of Neurosurgery	10.3109/02688697.2013.793292	No	No detailed information on cranial nerve palsies
16	2013	Chalouhi	Stroke	10.1161/STROKEAHA.113.001785	No	No detailed information on cranial nerve palsies
17	2013	De Vries	Stroke	10.1161/STROKEAHA.111.000434	No	No detailed information on cranial nerve palsies
18	2014	Buyukkaya	Interventional neuroradiology	10.15274/INR-2014-10070	No	No detailed information on cranial nerve palsies
19	2014	Moon	Journal of neurosurgery	10.3171/2014.7.JNS132677	Yes	
20	2014	Tanweer	AJNR. American journal of neuroradiology	10.3174/ajnr.A4081	Yes	
21	2014	Zanaty	Stroke	10.1161/STROKEAHA.114.006247	Yes	
22	2014	Heller	Journal of neurosurgery	10.3171/2014.3.JNS131493	No	Less than 10 patients with cranial nerve palsies treated by flow diversion
23	2014	Zhou	AJNR. American journal of neuroradiology	10.3174/ajnr.A3925	Yes	
24	2014	Moon	Neurological research	10.1179/1743132814Y.0000000322	No	No detailed information on cranial nerve palsies
25	2014	Puffer	AJNR. American journal of neuroradiology	10.3174/ajnr.A3826	Yes	
26	2014	Chalouhi	AJNR. American journal of neuroradiology	10.3174/ajnr.A3957	No	No detailed information on cranial nerve palsies
27	2015	Di Maria	AJNR. American journal of neuroradiology	10.3174/ajnr.A4437	No	No detailed information on cranial nerve palsies
28	2015	Sahlein	Journal of neurosurgery	10.3171/2014.12.JNS141777	Yes	
29	2015	Zanaty	Neurosurgery	10.1227/NEU.0000000000000607	Yes	
30	2015	Rouchaud	AJNR. American journal of neuroradiology	10.3174/ajnr.A4129	No	Less than 10 patients with cranial nerve palsies
31	2015	Wakhloo	AJNR. American journal of neuroradiology	10.3174/ajnr.A4078		At least 10 eligible patients, but insufficient information on clinical symptoms and follow-up
32	2015	Alghamdi	Expert Review of Medical Devices	10.1586/17434440.2015.1093413	No	Review article
33	2015	Fischer	AJNR. American journal of neuroradiology	10.3174/ajnr.A4420	No	No detailed information on cranial nerve palsies
34	2015	Shimizu	Acta neurochirurgica	10.1007/s00701-014-2251-1	No	No flow diverter devices applied
35	2015	Oh	Clinical neurology and neurosurgery	10.1016/j.clineuro.2014.11.008	No	Less than 10 patients with cranial nerve palsies treated by flow diversion
36	2015	Zhu	World neurosurgery	10.1016/j.wneu.2015.07.036	No	Review article
37	2016	Jevsek	Radiology and oncology	10.1515/raon-2016-0049		Less than 10 patients with cranial nerve palsies treated by flow diversion
38	2016	Kaya	Turkish neurosurgery	10.5137/1019-5149.JTN.14760-15.0	No	At least 10 eligible patients, but insufficient information on clinical symptoms and follow-up
39	2016	Breu	Radiology research and practice	10.1155/2016/2187275	Yes	
40	2016	Kim	Neurointervention	10.5469/neuroint.2016.11.1.10	Yes	
41	2016	Brown	Journal of neurosurgery	10.3171/2015.4.JNS142790	Yes	
42	2016	Durst	Journal of neurointerventional surgery	10.1136/neurintsurg-2015-011887	No	No detailed information on cranial nerve palsies
43	2016	Burrows	AJNR. American journal of neuroradiology	10.3174/ajnr.A4835	No	Less than 10 patients with cranial nerve palsies treated by flow diversion
44	2016	Kallmes	Interventional neurology	10.1159/000446503		No detailed information on cranial nerve palsies
45	2017	Miyachi	Neurointervention	10.5469/neuroint.2017.12.2.83	Yes	
46	2017	Bhogal	Frontiers in neurology	10.3389/fneur.2017.00381	No	No detailed information on cranial nerve palsies
47	2017	Griessenauer	Neurosurgery	10.1093/neuros/nyw110		At least 10 eligible patients, but insufficient information on clinical symptoms and follow-up
48	2017	Briganti	Journal of neurointerventional surgery	10.1136/neurintsurg-2016-012502		Less than 10 patients with cranial nerve palsies treated by flow diversion
49	2017	Peschillo	Operative Neurosurgery	10.1093/ons/opx032	No	No detailed information on cranial nerve palsies
50	2017	Miyachi	Journal of Stroke and Cerebrovascular Diseases	10.1016/j.jstrokecerebrovasdis.2016.12.023	No	Less than 10 patients with cranial nerve palsies treated by flow diversion
51	2017	Becske	Neurosurgery	10.1093/neuros/nyw014	No	No detailed information on cranial nerve palsies
52	2018	Silva	Journal of neurosurgery	10.3171/2018.1.JNS171774	Yes	
53	2018	ReXiaTi	Bio-medical materials and engineering	10.3233/BME-171718	No	Less than 10 patients with cranial nerve palsies treated by flow diversion
54	2018	Killer-Oberpfalzer	American Journal of Neuroradiology	10.3174/ajnr.A5592	No	At least 10 eligible patients, but insufficient information on clinical symptoms and follow-up
55	2018	Byvaltsev	Vestnik Rossiiskoi Akademii Meditsinskikh Nauk	10.15690/vramn918	No	Russian language
56	2018	Oishi	Neurologia Medico-Chirurgica	10.2176/nmc.oa.2018-0148	Yes	
57	2018	Pierot	Journal of neurointerventional surgery	10.1136/neurintsurg-2017-013559	No	No detailed information on cranial nerve palsies
58	2019	Sweid	World neurosurgery	10.1016/j.wneu.2019.07.115	No	No detailed information on cranial nerve palsies
59	2019	Yan	World neurosurgery	10.1016/j.wneu.2019.01.082	Yes	
60	2019	Pierot	Journal of neurointerventional surgery	10.1136/neurintsurg-2018-014261	No	No detailed information on cranial nerve palsies
61	2019	Kühn	Interventional neuroradiology	10.1177/1591019918792536	No	No detailed information on cranial nerve palsies
62	2019	Wang	Frontiers in Neurology	10.3389/fneur.2019.01191	Yes	
63	2019	Griessenauer	Neurosurgery	10.1093/neuros/nyy572	No	No detailed information on cranial nerve palsies
64	2019	Oğuz	Turkish Journal of Medical Sciences	10.3906/sag-1906-116	No	No detailed information on cranial nerve palsies
65	2019	Meyers	Stroke	10.1161/STROKEAHA.118.024135	No	No detailed information on cranial nerve palsies
66	2020	Nurminen	Clinical neurology and neurosurgery	10.1016/j.clineuro.2020.105782	No	Mixed patients with new (n=1) and existing (n=12) symptoms, not possible to differentiate
67	2020	Binh	Heliyon	10.1016/j.heliyon.2020.e03356	No	No detailed information on cranial nerve palsies
68	2020	Lv	The neuroradiology journal	10.1177/1971400919898109	No	Less than 10 patients with cranial nerve palsies treated by flow diversion
69	2020	Foreman	World neurosurgery	10.1016/j.wneu.2019.11.084	No	At least 10 eligible patients, but insufficient information on clinical symptoms and follow-up
70	2020	Daglioglu	Turkish neurosurgery	10.5137/1019-5149.JTN.25776-19.2	No	No detailed information on cranial nerve palsies
71	2020	Piano	Journal of Neurosurgery	10.3171/2019.1.JNS183005	No	At least 10 eligible patients, but insufficient information on clinical symptoms and follow-up
72	2021	Catapano	Journal of neurosurgery	10.3171/2021.5.JNS211149	No	No detailed information on cranial nerve palsies
73	2021	Lee	The neuroradiology journal	10.1177/19714009211013487	No	Less than 10 patients with cranial nerve palsies treated by flow diversion
74	2021	Kunert	Scientific reports	10.1038/s41598-021-87498-z		Less than 10 patients with cranial nerve palsies treated by flow diversion
75	2021	Link	Journal of clinical neuroscience : official journal of the	10.1016/j.jocn.2021.01.016	No	No detailed information on cranial nerve palsies
76	2021	Boulouis	Journal of neurointerventional surgery	10.1136/neurintsurg-2021-018188	Yes	
77	2022	Fehrenbach	Brain Sciences	10.3390/brainsci12030330	No	Less than 10 patients with cranial nerve palsies treated by flow diversion
78	2022	Fujii	Neurologia Medico-Chirurgica	10.2176/nmc.oa.2021-0203	Yes	
	2nd search on 22nd May:					
79	2022	Xu	Acta Neurochirurgica	10.1007/s00701-022-05239-1	Yes	
80	2022	Lee	AJNR. American journal of neuroradiology	10.3174/ajnr.A7498	Yes	

BMJ Publishing Group Limited (BMJ) disclaims all liability and responsibility arising from any reliance placed on this supplemental material which has been supplied by the author(s)

Supplemental Table 2: Patient demographics and aneurysm characteristics

Study	Patients with NOS[$] (%)	Visual symptoms only	Oculomotor symptoms only	Combined symptoms	Female (%)	Age (mean +/- SD)	Intradural aneurysms (%)	Extradural aneurysms (%)	Aneurysm size (mean +/- SD)
Yu et al[22]	14	0 (0%)	14 (100%)	0 (0%)	NR[#]	NR	NR	NR	<10 mm: 6; 10-25 mm: 4; >25 mm: 3
Szikora et al[28]	16	6 ((37.5%)	10 (62.5%)	0 (0%)	NR	NR	NR	NR	NR
O'Kelly et al[26]	36	12 (33.3%)	24 (66.7%)	0 (0%)	NR	NR	NR	NR	NR
Moon et al[35]	19	2 (10.5%)	17 (84.2%)	1 (5.3%)	14	65.8 +/- 14.5 years	5 (26.3%)	14 (73.7%)	17.7 +/- 7.8 mm
Tanweer et al[37]	19*	NR	NR	NR	NR	NR	0 (0%)	19 (100%)	NR
Zanaty et al[34]	51	0 (0%)	51 (100%)	0 (0%)	NR	NR	0 (0%)	51 (100%)	NR
Zhou et al[38]	11	4 (36.4%)	7 (63.6%)	0 (0%)	NR	NR	NR	NR	NR
Puffer et al[31]	24	1 (4.2%)	23 (85.8%)	0 (0%)	22 (91.7%)	73 +/- 10.1 years	0 (0%)	24 (100%)	22 +/- 4.6 mm
Sahlein et al[46]	39	13 (33.3%)	18 (46.2%)	8 (20.5%)	22 (56.4%)	58 +/- 10.8 years	14 (35.9%)[§]	24 (61.5%)[§]	22 +/- 5.9 mm
Zanaty et al[45]	12	12 (100%)	0 (0%)	0 (0%)	NR	NR	12 (100%)	0 (0%)	NR
Breu et al[49]	10	1 (10%)	5 (50%)	4 (40%)	10 (100%)	62.5 +/- 9.3 years	NR	NR	15.7 +/- 3.9 mm
Kim et al[53]	18	NR	NR	NR	NR	NR	NR	NR	NR
Brown et al[54]	45	10 (22.2%)	33 (73.3%)	2 (4.4%)	42 (93.3%)	64.7 years	19	26	18.6 mm
Miyachi et al[64]	18	0 (0%)	17 (94.4%)	1 (5.6%)	17 (94.4%)	70.8 +/- 11.8 years	0 (0%)	18 (100%)	21.7 +/- 7.1 mm
Silva et al[71]	15	15 (100%)	0 (0%)	0 (0%)	NR	NR	NR	NR	NR
Oishi et al[73]	38	10 (26.3%)	28 (73.7%)	0 (0%)	NR	NR	NR	NR	NR
Yan et al[79]	50	NR	NR	NR	NR	NR	NR	NR	NR
Wang et al[76]	22	15 (68.2%)	3 (13.6%)	4 (18.2%)	17 (77.3%)	53.5 +/- 11.4 years	NR	NR	10-25 mm: 11; >25 mm: 11
Boulouis et al[7]	54	15 (27.8%)	21 (38.9%)	18 (33.3%)	48 (88.9%)	59.2 +/- 15.9 years	33 (61.1%)	21 (38.9%)	16.2 +/- 7.6 mm
Fujii et al[93]	29	7 (24.1%)	22 (75.9%)	0 (0%)	NR	NR	NR	NR	NR
Xu et al[95]	26	26 (100%)	0 (0%)	0 (0%)	NR	NR	26 (100%)	0 (0%)	NR
Lee et al[94]	28	NR	NR	NR	NR	NR	NR	NR	NR

[$]NOS = neuro-ophthalmological symptoms induced by ICA aneurysm, treated with flow diversion

*Only data on cavernous aneurysms reported

'F/U = Follow-up

[#]Not reported

[§]Discrepancy in the manuscript

Supplemental Table 3: Neuro-ophthalmological outcomes in relation to time from symptom onset to treatment

	Within first month		Beyond first month	
**Study**	**Complete recovery**	**Improvement***	**Complete recovery**	**Improvement**
Brown et al[54]	NR[#]	11/11 (100%)	NR	12/27 (44.4%)
Wang et al[76]	NR	8/11 (72.7%)	NR	4/11 (36.4%)
Boulouis et al[7]	11/22 (50%)	19/22 (86.4%)	8/28 (28.6%)	18/28 (64.3%)

*Improvement = Complete & partial recovery
[#]Not reported

Supplemental Table 4: Visual and oculomotor outcomes

Study	Patients with NOS[$]	Visual: Visual symptoms only with F/U'	Visual: Complete recovery	Visual: Improvement	Oculomotor: Oculomotor symptoms only with F/U'	Oculomotor: Complete recovery	Oculomotor: Improvement
Yu et al[22]	14	0	-	-	13	10	13
Szikora et al[28]	16	6	3	5	10	7	10
O'Kelly et al[26]	36	9	1	5	18	11	13
Moon et al[35]	19	2	1	1	17	3	13
Tanweer et al[37]	19*	NR[#]	-	-	NR	-	-
Zanaty et al[34]	51	0	-	-	51	36	47
Zhou et al[38]	11	4	1	1	7	4	7
Puffer et al[31]	24	1	1	1	19	17	17
Sahlein et al[46]	39	13	0	7	22	1	14
Zanaty et al[45]	12	12	9	12	0	-	-
Breu et al[49]	10	1	0	0	5	0	5
Kim et al[53]	18	NR	-	-	NR	-	-
Brown et al[54]	45	10	NR	NR	33	NR	NR
Miyachi et al[64]	18	0	-	-	17	6	15
Silva et al[71]	15	15	NR	15	0	-	-
Oishi et al[73]	38	10	NR	3	28	NR	15
Yan et al[79]	50	NR	-	-	NR	-	-
Wang et al[76]	22	15	NR	7	3	NR	3
Boulouis et al[7]	54	14	0	5	20	5	13
Fujii et al[93]	29	7	NR	3	22	NR	17
Xu et al[95]	26	26	NR	20	0	-	-
Lee et al[94]	28	NR	-	-	NR	-	-

[$]NOS = neuro-ophthalmological symptoms induced by ICA aneurysm, treated with flow diversion
*Only data on cavernous aneurysms reported
'F/U = Follow-up
[#]Not reported

Supplemental Table 5: Complications and anatomical results

		Treatment-related complications		Anatomical result			
**Study**	**Patients**	**Morbidity**	**Mortality**	**Patient with anatomical F/U`**	**F/U' (mean +/- SD)**	**Complete occlusion**	**Incomplete occlusion**
Yu et al[22]	14	0	1	13	3.5 months (median)	7	6
Szikora et al[28]	16	NR	NR	NR	NR	NR	NR
O'Kelly et al[26]	36	NR	NR	NR	NR	NR	NR
Moon et al[35]	19	0	0	17	9.7 +/- 6.3 months	9	8
Tanweer et al[37]	19*	NR	NR	NR	NR	NR	NR
Zanaty et al[34]	51	NR	NR	NR	NR	NR	NR
Zhou et al[38]	11	0	0	NR	NR	NR	NR
Puffer et al[31]	24	0	0	20	10.3 +/- 7.6 months	14	6
Sahlein et al[46]	39	NR	NR	NR	NR	NR	NR
Zanaty et al[45]	12	0	0	NR	NR	NR	NR
Breu et al[49]	10	NR	1	8	NR	5	3
Kim et al[53]	18	NR	NR	NR	NR	NR	NR
Brown et al[54]	45	1	0	40	8.4 months (mean)	26	14
Miyachi et al[64]	18	0	0	18	6 months	11	7
Silva et al[71]	15	0	0	NR	NR	NR	NR
Oishi et al[73]	38	NR	NR	NR	NR	NR	NR
Yan et al[79]	50	NR	NR	NR	NR	NR	NR
Wang et al[76]	22	2	1	21	12.2 +/- 1.7 months	21	0
Boulouis et al[7]	54	4	2	50	13.3 +/. 10.5 months	37	13
Fujii et al[93]	29	NR	NR	NR	NR	NR	NR
Xu et al[95]	26	NR	NR	26	NR	23	3
Lee et al[94]	28	NR	NR	NR	NR	NR	NR

$NOS = neuro-ophthalmological symptoms induced by ICA aneurysm, treated with flow diversion

*PM = Prospective multi-center; PS = Prospective single-center; RS = Retrospective single-center; RM = Retrospective multi-center

'F/U = Follow-up

#Not reported

SUPPLEMENTAL FIGURES

PRISMA 2020 flow diagram for new systematic reviews which included searches of databases and registers only

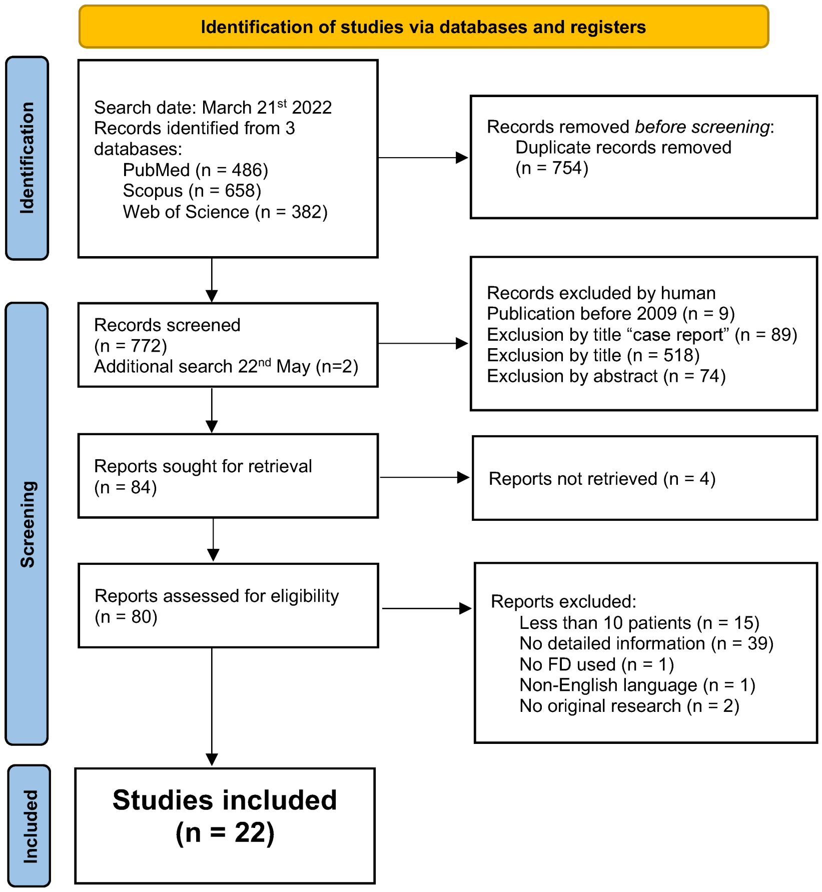

From: Page MJ, McKenzie JE, Bossuyt PM, Boutron I, Hoffmann TC, Mulrow CD, et al. The PRISMA 2020 statement: an updated guideline for reporting systematic reviews. BMJ 2021;372:n71. doi: 10.1136/bmj.n71

For more information, visit: http://www.prisma-statement.org/

Supplemental Fig. 1: Prisma flow diagram

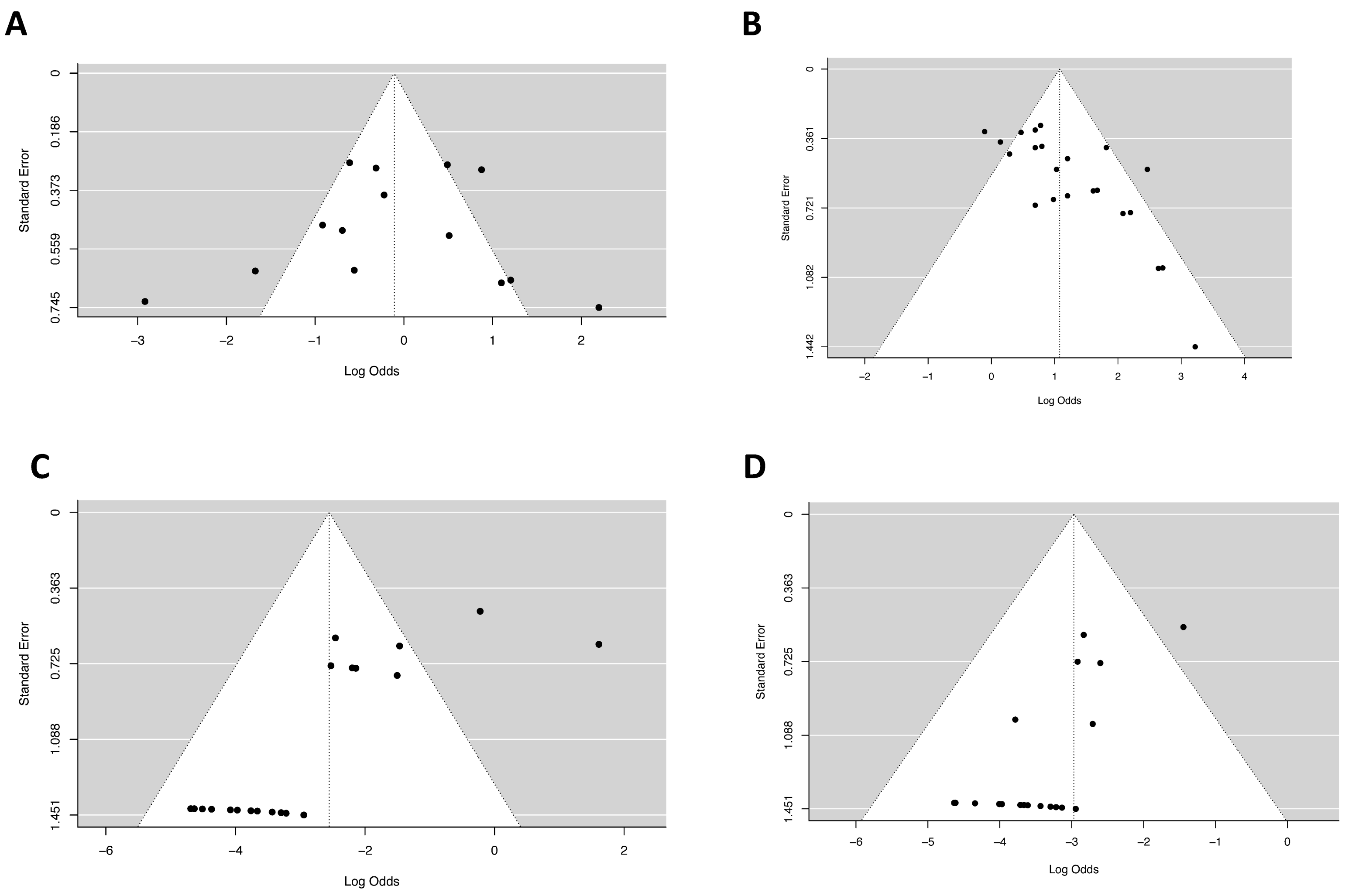

Supplemental Fig. 2: Funnel plots for complete recovery (A), improvement (B), transient (C) and permanent worsening (D).

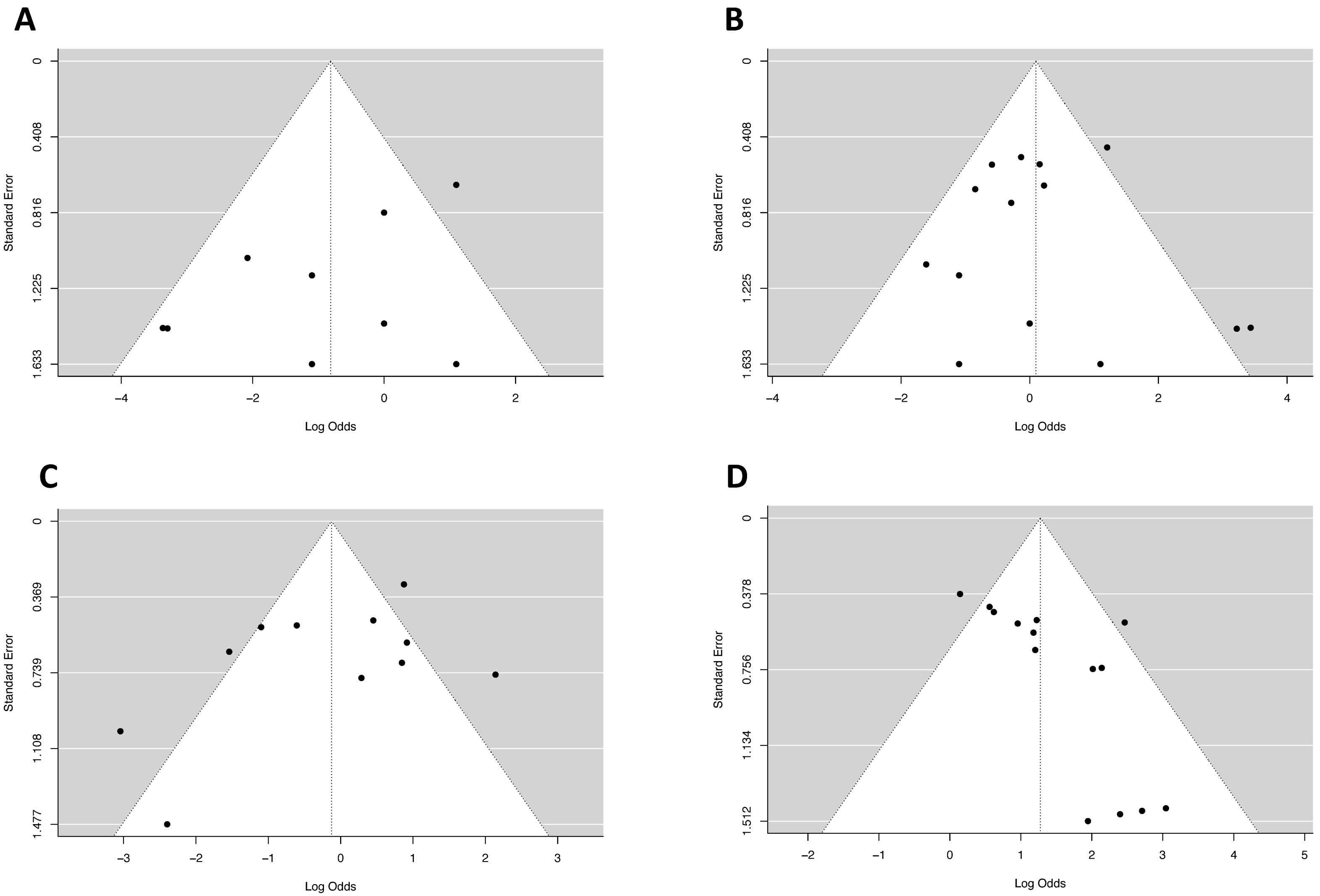

Supplemental Fig. 3: Funnels plots for complete visual recovery (A) and improvement (B) and on complete oculomotor recovery (C) and improvement (D).

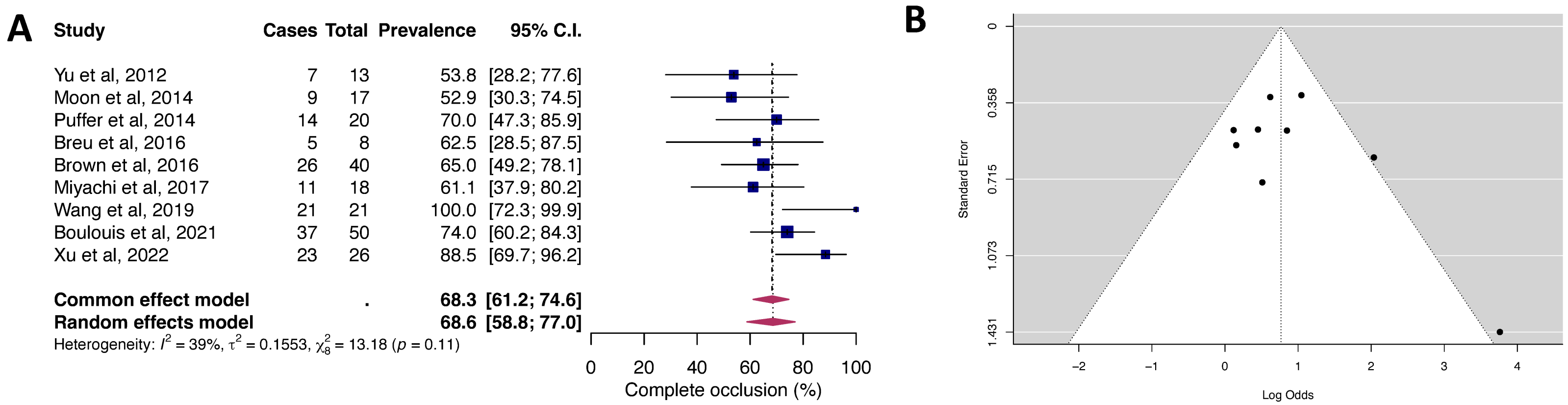

Supplemental Fig. 4: Forest plot (A) and corresponding funnel plot (B) for complete aneurysm occlusion.

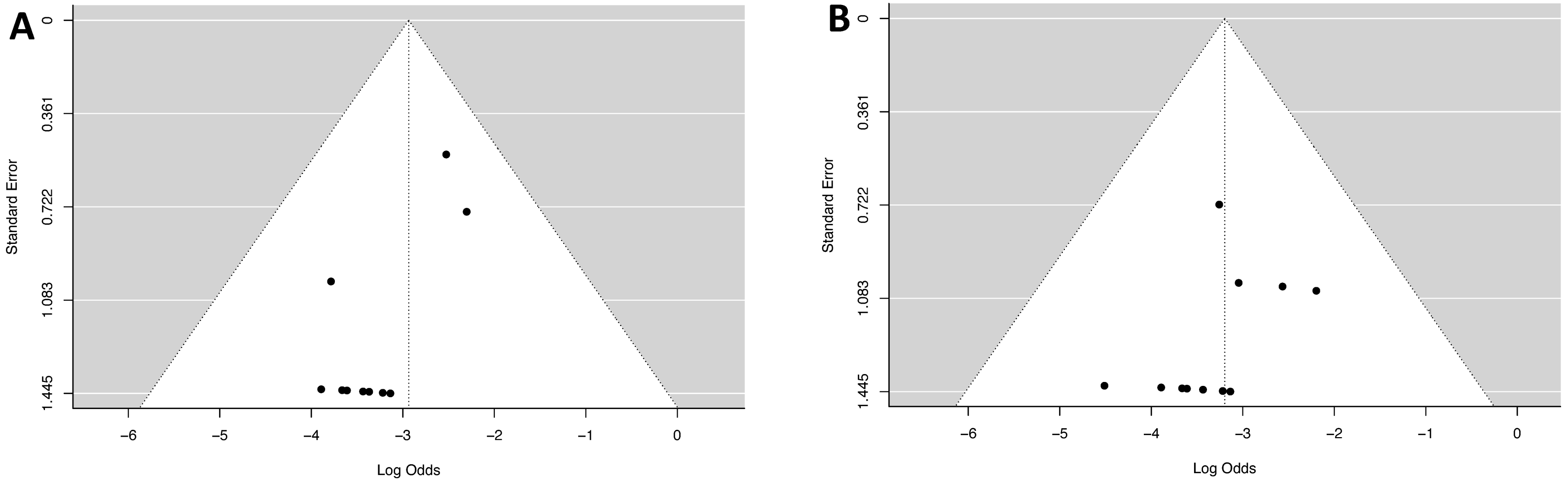

Supplemental Fig. 5: Funnel plots for morbidity (A) and mortality (B).

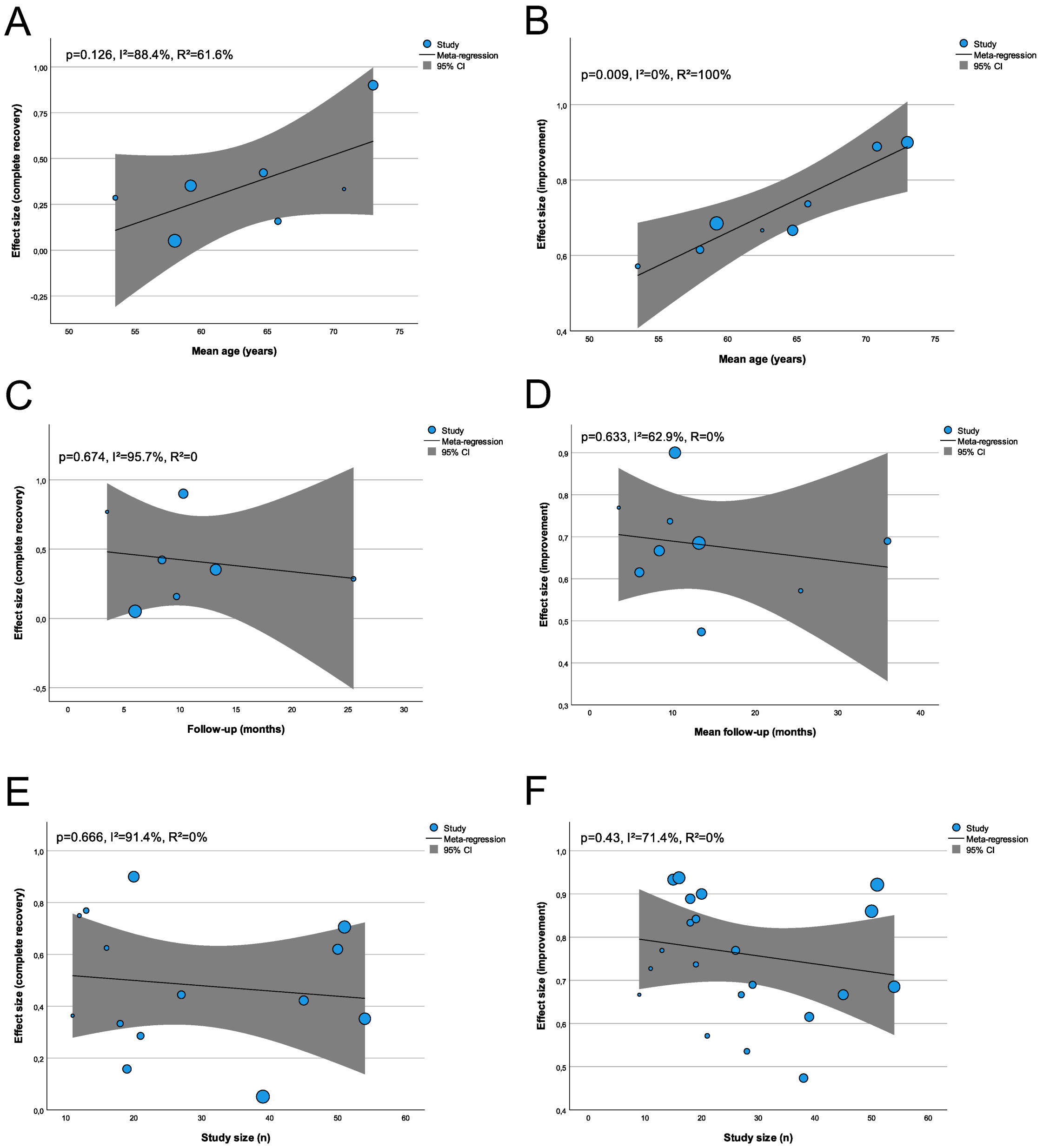

Supplemental Fig. 6: Bubble plots depicting the effect size (i.e. complete NOS recovery or improvement) in association with patient age (A, B), follow-up (C, D) and study size (E, F).
